# Promoter or inhibitor? The role played by housing prices on entrepreneurial vitality in China

**DOI:** 10.1371/journal.pone.0330660

**Published:** 2025-09-04

**Authors:** Yumei Guan, Chiwei Su, Yunfeng Wang

**Affiliations:** 1 School of Management, University of Sanya, Sanya, Hainan, China; 2 School of Economics, Qingdao University, Qingdao, China; 3 Faculty of Finance, City University of Macau, Macao, China; Universidade Federal do Tocantins, BRAZIL

## Abstract

This study delves into the time-varying causal relationship between housing prices (HP) and entrepreneurial vitality (EV) in China, employing a rolling-window sub-sample testing approach. The findings reveal that HP exerts both positive and negative influences on EV across different sub-samples. Specifically, when wealth effects dominate, HP positively impacts EV, acting as a promoter. Conversely, when substitution effects prevail, HP negatively affects EV, becoming an inhibitor. Additionally, EV consistently demonstrates a significant positive impact on HP across various sub-samples, indicating that EV serves as a predictor of HP trends. Given these complex and dynamic interactions, it is crucial for policymakers to carefully consider China’s entrepreneurial environment, financial constraints, and other relevant factors. Tailored policies for HP and EV should be designed based on distinct developmental stages to foster a balanced and sustainable economic environment.

## Introduction

This paper aims to answer the question of whether housing prices (HP) promote or inhibit entrepreneurial vitality (EV). Housing encompasses multiple attributes, including wealth storage, collateral, investment, and consumption [1]. For entrepreneurs, selling property can offer vital capital for entrepreneurial ventures [2]. Moreover, leveraging property as collateral enables access to bank financing [3]. Notably, as an investment asset, property’s favorable risk-return profile compared to entrepreneurial activities may significantly shape entrepreneurial decisions [4]. Entrepreneurial activities not only promote economic growth through knowledge spillover, technology diffusion, and demonstration effects [5], but also create social value by increasing employment and resident income [6]. Nowadays, EV has become one of the important factors influencing the potential for national development [7]. Therefore, countries around the world, especially developed economies, have elevated the promotion of entrepreneurship to a national strategic level. For example, the US has launched the “Startup America” initiative, which places entrepreneurial spirit as a core value and source of competitive advantage, providing comprehensive support for entrepreneurship. The EU has initiated the “2020 Entrepreneurship Action Plan” with the aim of fostering a new generation of entrepreneurs to revitalize the European economy. However, entrepreneurship is a high-investment and high-risk business activity [8]. Entrepreneurs must first overcome the capital threshold. Purchasing property or using it as collateral for a loan is a crucial way for entrepreneurs to secure funding [9,10]. Therefore, rising HP can increase the wealth levels of property owners, thereby increasing their startup capital and promoting entrepreneurial activities, generating a wealth effect for EV [11]. However, if HP rise, and the return on real estate investment exceeds that of entrepreneurship, some potential entrepreneurs may choose to invest in the property market rather than starting businesses, thus inhibiting entrepreneurial behavior and creating a substitution effect for EV [12]. Thus, the interaction between HP and EV is a complex and practically significant research topic.

In the past 20 years, China’s real estate industry has surged dramatically. The added value of China’s real estate sector increased from 2.3 trillion yuan in 2010 to 8.7 trillion yuan in 2024. At present, China has the largest real estate market in the world [13]. Additionally, from 2010 to 2024, the average annual growth rate of HP in China has exceeded 6%. China is one of the fastest-rising in HP globally [14]. For urban residents in China, housing is the largest asset for most households [15]. According to data from the People’s Bank of China, the homeownership rate for urban households reached 96% in 2023. Moreover, 41.5% of households own two or more properties in urban areas. Since 2010, China has also experienced a golden period for entrepreneurship [16]. A wide variety of highly active and high-growth market entities have continuously emerged [17]. From January 2010 to April 2024, the total number of new market entities in China reached 278 million. Among these, 32.6% are innovative startups primarily focused on Telecommunications, Media, and Technology. By the end of 2023, China had over 500 unicorn companies and thousands of IPO firms. The total valuation of China’s unicorns exceeds 13 trillion yuan, with major concentrations in fields such as hard technology, new energy, new media, digital economy, and artificial intelligence.

The above economic phenomena of HP and EV are highly overlapping in the history of China’s development. Additionally, the Chinese real estate market has distinct characteristics. First, from the perspective of collateral, due to the underdeveloped financial market in China, entrepreneurs face difficulties in financing [18], making household assets the primary source of funding in the early stages of entrepreneurship [19]. Housing, which constitutes 90% of Chinese household assets [20], is a key form of loan collateral and influences EV through wealth effects [21]. Second, from the investment perspective, the long-term upward trend in HP has led Chinese individuals to view housing as an investment with exceptionally stable returns, gaining significant investment favor. The real estate market is the most widely engaged investment for Chinese people, which can have a substitution effect on EV [22]. Third, from the consumption perspective, traditional Chinese culture values homeownership, viewing it as a prerequisite for marriage and childbirth [23,24]. Homeownership is also seen as an important indicator of social status. These cultural norms foster a strong preference for housing, which consumes a significant portion of household wealth, thereby limiting funds available for entrepreneurship. So, China provides a good case study for examining the relationship between HP and EV.

This study makes contribution in the following mode. First, existing research is mostly based on data from developed countries such as Europe and the US. This paper supplements the empirical evidence from developing countries, further enriching theories of HP and EV. Second, existing findings mainly focus on the micro-level, studying the impact and mechanisms of HP on entrepreneurial motivation, types, and intentions. This paper explores the interaction between HP and EV at the macro-level. This exploration can help the government formulate balanced policies for real estate development and entrepreneurial incentives, thereby achieving a positive interaction between the two. Third, existing studies primarily employ full-sample causality tests, which fail to delve into structural changes. In contrast, this paper utilizes a bootstrap sub-sample rolling-window causality test to explore the time-varying relationship between HP and EV in greater depth.

The structure of this paper is as follows: The literature review section synthesizes existing studies on HP and EV. The theoretical analysis section explores their relationship through the risk-return model. The methodology presents the rolling-window causality tests. The data section describes the dataset and variables. The empirical results section reveal time-varying HP-EV causal relationships. The conclusion section offers policy recommendations based on key findings.

### Literature review

Research on the relationship between HP and EV in developed countries has produced abundant evidence, with most empirical studies indicating a positive correlation between the two variables. Kerr et al. (2022) discover a strong correlation between HP growth and local entrepreneurship in the US, attributing it to the collateral channel [7]. Corradin & Popov (2015) find that an increase in home equity leads to an increase in the transition of individuals into self-employment [25]. Hurst & Lusardi (2004) observe that regions with higher HP appreciation have a higher likelihood of new business startups [26]. Fairlie and Krashinsky (2012) find that real estate appreciation increases the probability of entrepreneurship in the US [27]. Reuschke (2016) highlights the facilitation of home-based self-employment by housing wealth in the UK [1]. Adelino et al. (2015) demonstrate that rising HP leads to a significant increase in small business starts through the collateral lending channel in the US [2]. Da Fonseca & Pannella (2023) note immediate positive effects on entrepreneurship during a housing boom in the US [5]. Disney & Gathergood (2009) identify a positive relationship between household wealth and self-employment entry in the UK, suggesting a link between entrepreneurial preferences and household wealth [28]. Jensen et al. (2022) find that housing collateral enabled individuals with higher abilities and less-established track records to overcome credit constraints and start new firms in Denmark [6].

There are also studies that present opposite perspectives. Kacher & Petach (2021) argue that higher HP leads to a decrease in new establishment openings relative to existing ones, indicating a potential crowding-out effect [12]. Gholipour (2020) finds a significant negative impact of real HP increases on small and medium-sized industrial entrepreneurship in Iran [29]. Additionally, some scholars contend that HP has no discernible effect on entrepreneurship. Berggren et al. (2020) suggest that stable high HP do not influence entrepreneurship in Sweden. Others argue that the influence of housing prices on entrepreneurship is nuanced [30]. Bracke et al. (2018) indicate that the relationship between housing equity and entrepreneurship is complex and could vary, due to competing portfolio and wealth effects [31]. Harding & Rosenthal (2017) find that the connection between homeownership and self-employment is more pronounced during periods of rapid housing price growth but less significant when housing capital gains are limited or negative [32].

Recently, researchers have increasingly focused on examining the influence of HP and EV in China, with some studies highlighting a positive impact. Wang (2012) suggests that increased housing wealth enables households to increase entrepreneurship activity by overcoming credit constraints [33]. Oh et al. (2021) observe that higher HP growth rates correlate with a greater likelihood of family members being self-employed [33]. Fan et al. (2023) demonstrate that rising HP stimulates entrepreneurial activity through both the wealth effect and the siphon effect [35]. Huang et al. (2023) find that households with greater investments in housing are more inclined to initiate new businesses, especially those owning their homes outright [36]. Luo (2019) indicates that family wealth significantly boosts entrepreneurial tendencies, particularly in regions with lower GDP per capita [37]. Liu & Zhang (2021) quantify that every 10,000 yuan increase in housing wealth increases the probability of household business ownership by approximately 0.7 percentage points [38]. Chen & Hu (2019) find that the relationship between homeownership and entrepreneurial engagement varies with housing values, showing increased entrepreneurial participation as housing values rise [39]. On the contrary, there are studies that present opposing viewpoints. Li & Wu (2014) discover that high HP generally deters entrepreneurial activities among urban adults [40].

Furthermore, some scholars argue that the relationship between HP and EV is nonlinear and intricate. Wang & Hu (2023) identify an inverted U-shaped pattern in the association between HP and urban entrepreneurship [41]. Hu & Qian (2022) find that high urban HP has a significantly negative impact on the inclination of urban individuals to engage in necessity-driven entrepreneurship, while showing no significant influence on opportunity-driven entrepreneurship [42]. Liu et al. (2022) demonstrate that an increase in HP generally encourages family entrepreneurial decision-making, with a more pronounced effect for families with housing property rights. Conversely, for families without housing property rights, the rise in HP has an inhibitory effect on family entrepreneurship [43].

In short, in recent years, scholars have begun to explore the relationship between HP and EV. However, evidence based on developed countries is abundant, and empirical evidence from developing countries is lacking. In addition, the existing empirical research is basically linear correlation results, lacking evidence of complex and dynamic relationships.

### Risk-return model

**Fig 1:**
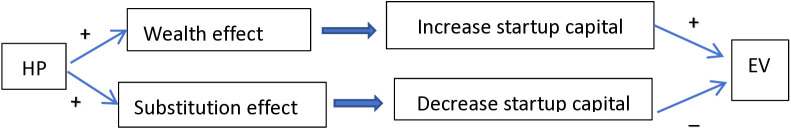
The impact mechanism of HP on EV.

HP has a mixed influence on EV, which is illustrated in Fig 1. HP affects EV through two channels. The first is the wealth effect channel [1,5–7,25,34]. In this channel, an increase in HP allows entrepreneurs to generate more entrepreneurial funding by selling their properties or securing larger loans through mortgaging their properties, subsequently having a positive impact on EV. The second is the substitution effect channel [12,29,40,41]. An increase in HP results in higher investment returns and decreased risk in real estate, thereby attracting more investment. Thus, the increase in HP may cause real estate investment to act as a substitute for entrepreneurial investment, which has a negative impact on EV. As a result, the impact of HP on EV is ambiguous and complicated.

The substitution effect channel from HP to EV, can be specifically explained by the risk-return model. The risk-return model, introduced by Markowitz (1952), emphasizes the importance of portfolio diversification [44,45]. If investors allocate their investments between real estate and start-ups, with expected returns of $E(r_{1})$ and $E(r_{2})$, respectively, and variances of $\sigma_{1}^{2}$ and $\sigma_{2}^{2}$ for real estate and start-ups, respectively, along with a covariance $Cov(r_{1},r_{2})=\rho \sigma_{1}\sigma_{2}$ between the two investments, where ρ is the correlation coefficient, they must consider both risk and return when investing in real estate. Additionally, if the proportion of investment in real estate and start-ups is $w_{1}$ and $w_{2}$, respectively, investors aim to maximize the expected portfolio return $E(r_{p})=w_{1}E(r_{1})+w_{2}E(r_{2})$ under a certain level of risk $Var(r_{p})=w_{1}^{2}\sigma_{1}^{2}+w_{2}^{2}\sigma_{2}^{2}+2w_{1}w_{2}Cov(r_{1},r_{2}).$ The optimization problem for the investor can be formulated as follows:


$$MaxE(r_{p})=w_{1}E(r_{1})+(1-w_{1})E(r_{2})$$

S.t. $w_{1}^{2}\sigma_{1}^{2}+(1-w_{1}{)}^{2}\sigma_{2}^{2}+2w_{1}(1-w_{1}rho\sigma_{1}\sigma_{2}=\sigma^{2}$, where $\sigma^{2}$ is given.

The resulting first order conditions of the Lagrange function can be expressed as:


$$\frac{\partial L}{\partial w_{1}}=E(r_{1})-E(r_{2})+2\lambda [w_{1}\sigma_{1}^{2}-(1-w_{1})\sigma_{2}^{2}+(1-2w_{1})\rho \sigma_{1}\sigma_{2}]=0$$ (1)

Equation 1 indicates the relationship between $w_{1}$ and $\sigma_{1}$. Then we have:


$$\frac{\partial w_{1}}{\partial \sigma_{1}}=-\frac{\rho \sigma_{2}+2w_{1}(\sigma_{1}-\rho \sigma_{2})}{\sigma_{1}^{2}+\sigma_{2}^{2}-2\rho \sigma_{1}\sigma_{2}}$$

As $\rho <1,\sigma_{1}>\sigma_{2}$, then $\frac{\partial w_{1}}{\partial \sigma_{1}}<0$. The result above can be used to explain the substitution effect channel from HP to EV.

## Methodology

### Bootstrap full-sample causality test

Granger causality tests relies on stationary time series for valid inference. When this assumption is violated, traditional VAR models may produce spurious results [46]. Shukur & Mantalos (2000) developed the residual bootstrap (*RB*) method to significantly improve the accuracy of Granger causality testing [47]. Their likelihood ratio (*LR*) approaches, as discussed in Shukur & Mantalos (2004), enhance test power and size properties, particularly for small sample sizes [48]. To rigorously examine the causal relationship between HP and EV, this study employs a modified likelihood ratio statistic for testing. The bivariate VAR (*p*) process is s*p*ecified by Equation (2):


$$Y_{t}=a_{0}+a_{1}Y_{t-1}+\ldots +a_{p}Y_{t-p}+\varepsilon_{t},t=1,2,3\ldots \ldots T$$ (2)

where p is chosen using the Schwarz Information Criterion (SIC) to determine the optimal lag order. Additionally, Y can be further expressed as $Y_{t}=(HP_{t},EV_{t})\prime$.. Moreover, because financing constraints (FC) can simultaneously influence both HP and EV [49–52], they may confound the observed relationship between these variables. Therefore, in our VAR framework, we incorporate FC as a control variable, leading to the following modified specification of Equation (2):


$$\begin{array}{c} \left[\;\;\begin{array}{c} {}*20lHP_{t}\\ EV_{t} \end{array}\;\;\right]=\left[\;\;\begin{array}{c} {}*20ca_{10}\\ a_{20} \end{array}\;\;\right]+\left[\;\;\begin{array}{cc} {}*20la_{11}(L) & a_{12}(L)\begin{array}{cc} {}*20l & a_{13}(L) \end{array}\\ a_{21}(l) & a_{22}(L)\begin{array}{cc} {}*20l & a_{23}(L) \end{array} \end{array}\;\;\;\;\right]\begin{array}{c} {}*20c \end{array}\left[\;\;\;\;\begin{array}{c} {}*20cHP_{t}\\ \begin{array}{c} EV_{t}\\ FC_{t} \end{array}\;\;\;\; \end{array}\right]+\left[\;\;\begin{array}{c} {}*20c\varepsilon_{1t}\\ \varepsilon_{2t} \end{array}\;\;\right] \end{array}$$ (3)

where $\varepsilon_{t}=(\varepsilon_{1t},\varepsilon_{2t})\prime$ represents a white-noise process. $a_{ij}(L)=\sum {\mathrm{\backslash nolimits}}_{k=1}^{p}a_{ij,k}L^{k},i,j=1,2,$ and L signifies a lag operator, that is $L^{k}Y_{t}=Y_{t-k}$. We can analyze Equation (3) on account of $a_{12,k}=0$, where *k* =1,2,......*p*. Rejecting the null hypothesis that HP does not cause EV suggests that HP can influence EV. Likewise, we can examine the null hypothesis that EV does not impact HP using Equation (3).

### Parameter stability test

The bootstrap full-sample causality test assumes that VAR models have constant parameters, disregarding potential structural changes that may arise in real-world situations. Parameter instability could reduce the accuracy of test results. To address this issue and mitigate the impact of parameter structural changes on test outcomes, this research employs Sup−F,Ave−F and Exp−F tests [53,54] to evaluate parameter stability. Furthermore, the $L_{c}$ statistic developed by Nyblom (1989) and Hansen (1992) is utilized to determine whether the parameters follow a random walk process and assess their long-term stability [55,56]. If the parameters exhibit instability, it indicates that the causal relationship between HP and EV varies over time. Accordingly, a sub-sample test is carried out to explore the causal connection between HP and EV, consistent with the earlier discussion.

### Bootstrap sub-sample rolling-window causality test

In addressing the issue of parameter structural changes, we employ the sub-sample rolling-window causality test introduced by Balcilar et al. (2010) [57]. This method entails dividing the complete sample into several sub-samples with a fixed window width (*f*). With a sequence length of *E*, the original sample is segmented into E−f sub-samples. Subsequently, an improved *LR* test utilizing the *RB* method is conducted on each of these sub-samples. By examining the *LR* statistics and associated *p*-values of the E−f sub-samples, we can determine the causal relationship between HP and EV. $N_{b}^{-1}\sum_{k=1}^{p}{\overset{\wedge }{\underset{21,k}{a}}}^{*}$ and $N_{b}^{-1}\sum_{k=1}^{p}{\overset{\wedge }{\underset{21,k}{a}}}^{*}$ indicate the impact of HP on EV and the effect of EV on HP, respectively. $N_{b}$ represents the frequency of bootstrap repetitions. ${\overset{\wedge }{\underset{12,k}{a}}}^{*}$ and ${\overset{\wedge }{\underset{21,k}{a}}}^{*}$ are parameters derived from Equation (3). In this study, we employ a 90% confidence interval, along with their respective lower and upper bounds, corresponding to the 5th and 95th quantiles of ${\overset{\wedge }{\underset{12,k}{a}}}^{*}$ and ${\overset{\wedge }{\underset{21,k}{a}}}^{*}$, respectively.

### Data and descriptive analysis

In this paper, the monthly data from 2010M01 to 2024M04 are selected to examine the causal nexus between HP and EV. In 2010, China’s “4-Trillion-Package” in response to the global financial crisis yielded visible results. The Chinese market rebounded, and investor and consumer confidence were restored. China’s GDP reached 5.879 trillion US dollars, a 10.3% increase, surpassing Japan to become the world’s second-largest economy. China’s industrial scale exceeded that of the United States, making it the world’s largest. Rapid economic development and wealth accumulation have led to strong housing demand, resulting in a long-term housing boom. Additionally, since 2010, China has implemented a series of policies to support and encourage entrepreneurship **to** help small and medium-sized enterprises survive the hardships of the global financial crisis. These policies cover aspects such as entrepreneurial education, startup capital, and entrepreneurial taxation, providing various incentives and support for startups. The national encouragement of entrepreneurship has garnered more societal recognition and individual preference for entrepreneurship. The related policy incentives and support have reduced the barriers and risks of entrepreneurship, promoting EV. In summary, a favorable entrepreneurial environment has been established. Therefore, we chose January 2010 as the starting point for our research period.

We obtained the monthly average selling price of housing to reflect the level of HP, with the data sourced from the National Bureau of Statistics of China. Additionally, we selected the quantity of newly registered business entities to measure EV. The number of newly registered business entities is calculated as the sum of newly registered companies and newly registered individual businesses, obtained from the China State Administration for Market Regulation. Furthermore, financing constraints (FC) are an important determinant of entrepreneurship [8]. Particularly in emerging market countries, where many entrepreneurial activities, face challenges due to underdeveloped financial markets and relatively low capital stock [51,52]. As the financial markets develop, entrepreneurs can obtain financing support through loans to engage in entrepreneurial activities [10]. In addition, when banks shrink the scale of commercial credit, debt financing for homebuyers will be limited. As a result, the housing demand been restricted [49,50]. Therefore, banks affect HP, EV and other economic activities by providing loans [58]. We select the loan growth of financial institutions to measure FC. The data for FC come from official website of the People’s Bank of China.

**Fig 2:**
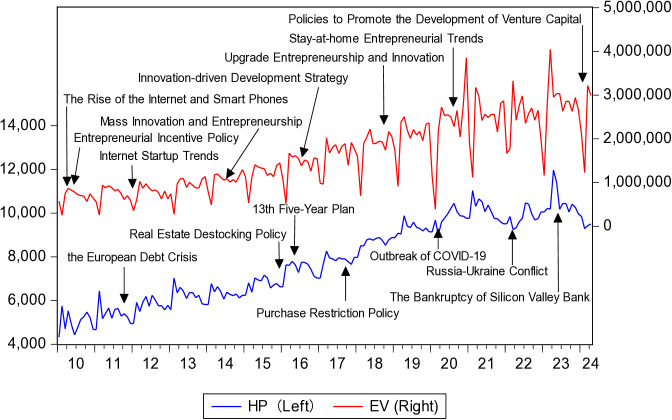
The trend of HP and EV.

Fig 2 shows the trend of HP and EV. It can be observed that the long-term EV shows an upward trend across the sample period. In 2010, the iPhone 4 entered China. Smartphones, 4G networks and a large market have given rise to huge opportunities for Internet entrepreneurship in China. In China, entrepreneurs believe that all industries are worth doing over again with the mobile Internet. Education, finance, media, retail, fresh food, catering, leasing, takeout, public benefit and other fields are full of various entrepreneurial projects. In addition, since 2010, the Chinese government has implemented policies to encourage entrepreneurship. It has increased support for small enterprises, individual businesses, and especially new enterprises. Thus, China’s entrepreneurial environment has been constantly improving, and the public’s entrepreneurial aspirations have been continuously rising. In 2014, China saw an average of 10,400 new businesses registered every day, adding 27.54 million individual businesses within a year. Over the following decade, different entrepreneurial trends emerged each year. Even during the severely impacted year of 2020, which was marked by the COVID-19 pandemic, China’s stay-at-home economy entrepreneurial trends, such as online education, cloud office, online grocery shopping, and telemedicine, provided numerous opportunities for entrepreneurship. The number of newly added business entities in China grew from 8.12 million in 2010 to 336 million in 2023, with an average annual growth rate between 10% and 30%. However, during the research period, HP experienced several significant fluctuations. There was a notable decrease in 2012 due to the impact of the European debt crisis. At the end of 2015, the central government officially implemented the de-stocking policy for the real estate market. The effects of this policy quickly became apparent in the first half of 2016, with both transaction volumes and prices in the real estate market substantially rising. This prosperous state continued until 2020. When the COVID-19 pandemic broke out in 2020, HP declined. After that, HP was affected by the Russia-Ukraine conflict, experiencing a significant drop in February 2022. In March 2023, it dropped again due to the bankruptcy of Silicon Valley Bank in the United States.

In short, China has been seriously affected by the global complex and serious situation. By comparing the shocks and trends in HP and EV, we find that these two variables sometimes change in the same direction, and sometimes in the opposite direction. Overall, the relationship between HP and EV is changeable. Hence, it is reasonable to utilize the sub-sample technique to analyze this dynamic and complex interaction.

Table 1 shows descriptive statistics. The mean values of HP, EV and FC are 7818.340, 1614005

**Table 1:** Descriptive statistics.

	Mean	Median	Maximum	Minimum	Standard Deviation	Skewness	Kurtosis	Jarque-Bera
HP	7818.340	7768.770	11970.28	4326.020	1868.731	−0.002	1.712	11.886^***^
EV	1614005	1464054	4045187	253643	826483.2	0.473	2.327	9.656^***^
FC	1221829	1103140	2477818	413679.6	602364.0	0.476	2.010	13.469^***^

Notes: ^***^ denotes significance at the 1% level.

and 1221829, respectively. The maximum value and minimum value of the three variables, vary considerably. This means they are highly volatile. The skewness is negative in HP and positive in EV and FC. The kurtosis values of HP, EV and FC are lower than 3, follow a platykurtic distribution. In addition, the statistics of the Jarque-Bera test for three variables are significant at the 1% level. thus, they followed a nonnormal distribution. Hence, the traditional Granger causality test is not reasonable to apply.

## Empirical results

The VAR model, selecting the optimal lag length of 3 according to the SIC, is used to analyze the full-sample causality between HP and EV. Table 2 displays the results of the full-sample Granger causality test. It indicates that HP Granger-causes EV, consonant with the existing literature [5,7,12,25,35,36]. However, EV does not Granger-cause HP. Nevertheless, due to structural variations causing instability in the parameters, the causality results may be unreliable in the full sample. To address this issue, tests for parameter stability were conducted, including the *Sup-F, Ave-F, Exp-F* and *Lc* tests [53,54]. In statistical testing, although the null hypotheses of the first three tests are identical, their alternative hypotheses exhibit significant differences [54]. The *Sup-F* test is designed to detect abrupt structural changes, while the *Mean-F* and *Exp-F* tests focus on gradual parameter variations over time. Table 3 presents the corresponding test results. The *Sup-F* test results indicate that, at the 1% significance level, both the two series and the VAR system exhibit significant abrupt structural breaks. The *Mean-F* and *Exp-F* tests reveal that, at the 1% significance level, these systems demonstrate gradual parameter evolution over time. Additionally, at the 1% significance level, the *Lc* test further confirms the non-constancy of parameters across the entire VAR system. Integrating all test results, we conclude that the parameter instability in the full-sample VAR model indeed stems from structural changes in the data. This instability in causal linkages between HP and EV, led to the use of a bootstrap sub-sample rolling-window causality test to further explore their relationship. The rolling sub-sample data include 24 months of observations to ensure the reliability of the test [48,59]. To verify the reliability of causation, we also examined widths of 20, 28, and 32 months, which yielded results that were largely consistent with those of 24 months.

**Table 2:** Full-sample Granger causality tests.

Tests	H0: HP does not Granger cause EV		H0: EV does not Granger cause HP	
	Statistics	*p*-values	Statistics	*p*-values
Bootstrap *LR* test	40.581	0.000	4.061	0.420

Notes: *p*-values are calculated from 10,000 bootstrap repetitions.

**Table 3:** The parameter stability test.

Tests	HP		EV		VAR system	
	Statistics	*p*-value	Statistics	*p*-value	Statistics	*p*-value
*Sup-F*	38.156^***^	0.000	125.013^***^	0.000	95.139^***^	0.000
*Ave-F*	20.316^***^	0.000	47.940^***^	0.000	45.823^***^	0.000
*Exp-F*	15.426^***^	0.000	57.716^***^	0.000	43.145^***^	0.000
*Lc*					0.988^***^	0.000

Notes: *p*-values are calculated from 10,000 bootstrap repetitions.

*** represent significance at 1% levels.

**Fig 3:**
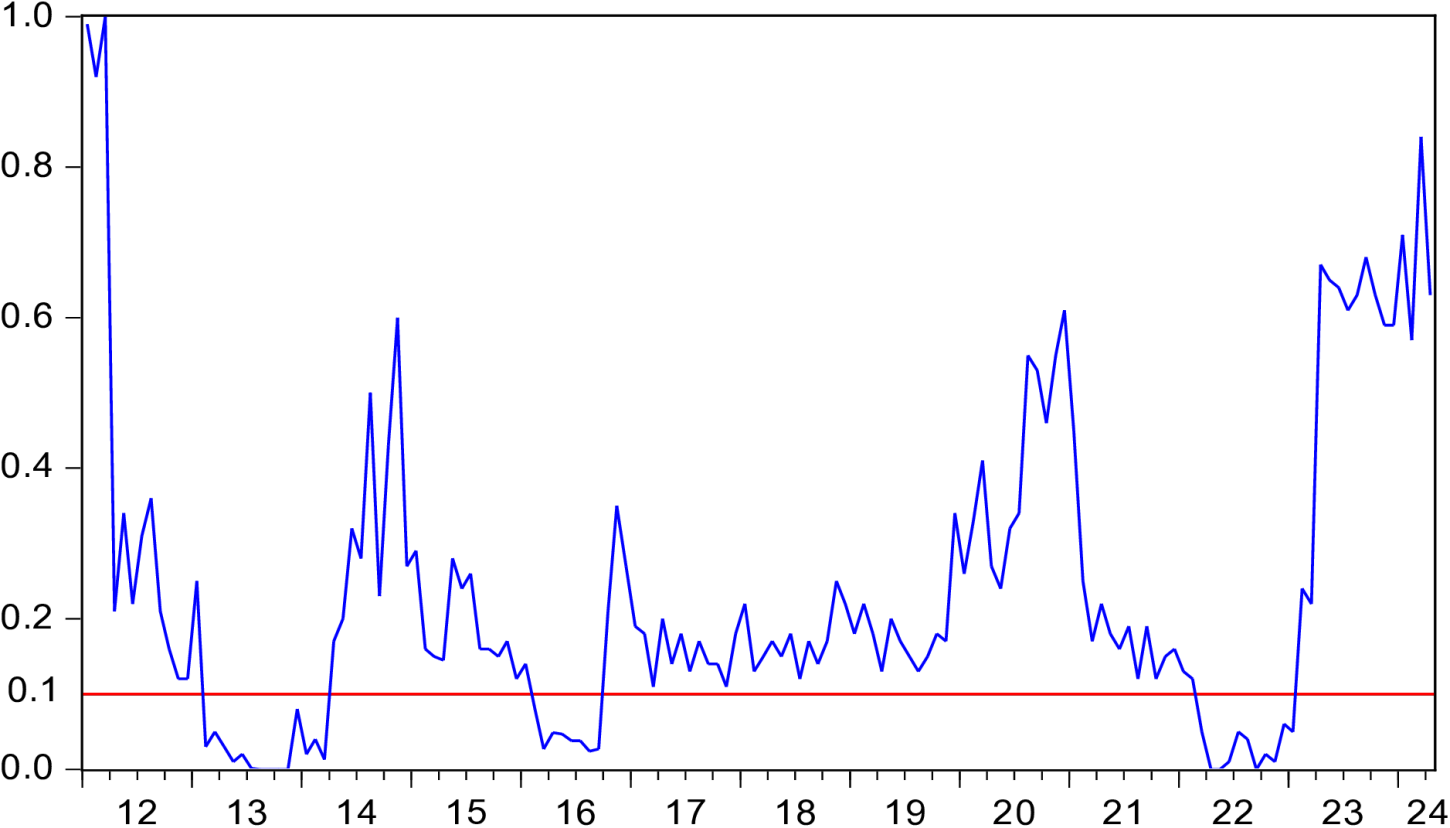
*p*-values of the rolling-window estimation examining the null that HP is not Granger cause of EV.

**Fig 4:**
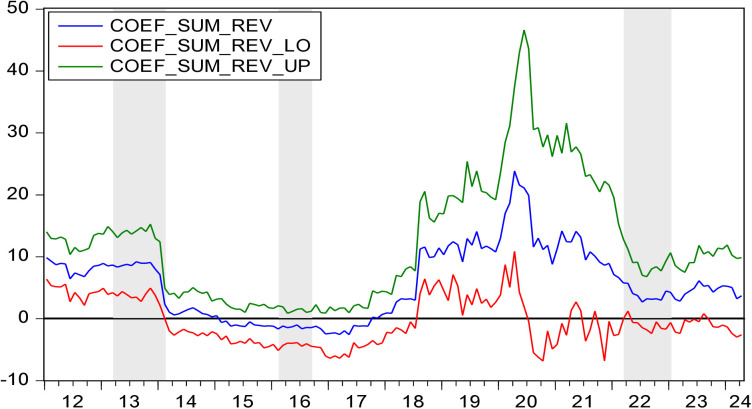
The coefficients for the effect of HP on EV.

Fig 3 presents *p*-values for the hypothesis that HP does not Granger cause EV. Fig 4 illustrates the direction of influence from HP to EV. Figs 3 and 4 reveal that during the periods 2013M02-2014M03 and 2022M03-2023M01, HP has a positive impact on EV. During the periods of 2016M02-2016M09,HP has a negative impact on EV.

During the periods of 2013M02-2014M03, HP showed an upward trend. In February 2013, the Chinese State Council reiterated policies such as purchase restrictions and loan restrictions to crack down on speculative home buying. However, HP did not decrease but instead increased. From M02 to M08 in 2013, HP in 70 major Chinese cities all rose, with 47 of them experiencing increases of over 10%. Additionally, in response to the European debt crisis, China implemented a series of measures to address the significant operational pressures, rising costs, financing difficulties, and heavy taxes and fees faced by small and micro-sized entrepreneurial enterprises, and these measures yielded positive results. In 2013, the number of newly registered enterprises in China reached 110 million, an increase of 28.64%. The positive impact of HP on EV can be explained from three aspects. First, HP rises and housing wealth increases. By monetizing housing wealth, more self-owned funds can be obtained, thus boosting EV [35]. Second, HP rises and the value of housing collateral improves. Mortgaging the house to the bank allows entrepreneurs to get more mortgage loans [60]. As a result, the FC of entrepreneurship decreases and EV increases [21,61,62]. Third, rising HP and increasing housing wealth can enhance individuals’ expectations of investment ability and risk tolerance. This leads to an enhanced preference for entrepreneurship and an increased EV [36].

During the periods of 2016M02-2016M09, HP showed an upward trend. In 2016, after suffering from excessive development in the previous two years, resolving the inventory of the real estate was a significant task for China’s economic development. The government implementing a series of measures to reduce inventory, including lowering interest rates, easing loan application thresholds, reducing transaction taxes, increasing leverage for home purchase credit, and other policies to encourage residential property purchases. These stimulus measures led to a 42.1% increase in property sales in the first half of 2016. The real estate market experienced a significant increase in both volume and price. China’s first-tier cities experienced dramatic HP surges. Shenzhen’s HP surged from 20,000 yuan per square meter in early 2013 to 51,000 yuan by June 2016, marking a 155% increase over three years, with nearly half of that growth occurring in the first half of 2016 alone. Shanghai saw HP climb from 25,000 yuan to over 40,000 yuan per square meter during the same period, a 60% cumulative increase that included a 33.5% spike in the first six months of 2016. This rapid price appreciation fueled speculation in real estate markets, diverting substantial capital away from productive sectors and entrepreneurial investments. While annual household incomes in these cities grew at 5% to 7% from 2013 to 2016, HP increases ranging from 25% to 155% severely distorted housing affordability. By 2016, Shenzhen’s price-to-income ratio reached an alarming 34:1, with Beijing and Shanghai both exceeding 25:1. These figures dwarfed the internationally recognized warning threshold of 6:1, effectively pricing ordinary wage earners out of homeownership and imposing severe financial burdens even on middle-class households. In addition, China’s household leverage ratio rose from approximately 30% in early 2013 to 44.8% by 2016, approaching the International Monetary Fund’s 50% risk threshold. New mortgage loans jumping 111% year-over-year to 2.36 trillion yuan in the first half of 2016, accounting for over 50% of total new loans. This contrasted sharply with the 25% share and 1 trillion yuan volume recorded in 2013. The reallocation of credit from productive economic activities to real estate speculation had substantial crowding-out effects on entrepreneurial financing. The wealth effect from housing appreciation was ultimately replaced by debt servicing burdens, constraining entrepreneurial activity. In 2016, the number of newly registered market entities in China reached 16.51 million, with a growth rate of 11.6%, marking the lowest growth rate since 2013. During this period, the negative impact of HP on EV can be explained from three aspects. First, there is a substitution relationship between entrepreneurial investment and real estate investment. As HP rises, the return on investment in real estate exceeds that of entrepreneurial investment, leading to a significant influx of funds into the real estate market. The increase in real estate investment results in a reduction in entrepreneurial funds, creating a substitution effect on entrepreneurial investment [22,40]. Second, the rise in HP increases entrepreneurial costs such as wages and rents, reducing entrepreneurial profits and increasing the likelihood of entrepreneurial failure, thereby weakening individuals’ entrepreneurial intentions and leading to a decline in EV [42]. Third, the rise in HP forces people to spend more on purchasing houses and bear heavier mortgage loan. This makes people more inclined to choose a stable-income jobs. Entrepreneurship requires taking on enormous risks and uncertainties [63]. Therefore, rising HP will limit entrepreneurial risk-taking and lead to lower EV [31].

During the periods of 2022M03-2023M01, HP showed a downward trend. In March 2022, the Russia-Ukraine conflict erupted. As a result, China’s economy, which had not yet recovered from the shadow of COVID-19, was negatively affected again. Consumers became more pessimistic about the future and reduced their consumption significantly. These factors affected the real estate market. In 2022, real estate sales amounted to 1333 billion yuan, representing a decrease of 26.7%. HP also dropped noticeably. In addition, the war exerted pressure on global supply chains, causing oil and grain prices to rise sharply. China is the world’s largest importer of oil and grain. Inflation led to an increase in the price of production factors such as raw materials and labor, which increased the cost of running enterprises and worsened the entrepreneurial environment, resulting in fewer entrepreneurial activities [64]. In 2021, the number of newly registered market entities in China was 29,604,140, while in 2022, it was 29,644,429, showing almost no growth. The positive impact of HP on EV can be explained from two aspects. First, the decrease in HP leads to a reduction in housing wealth, thereby weakening the ability to withstand investment risks and lowering EV [41]. Second, the decrease in HP results in fewer funds from mortgage loans [25,65], leading to a decrease in entrepreneurial capital and thus reducing EV.

**Fig 5:**
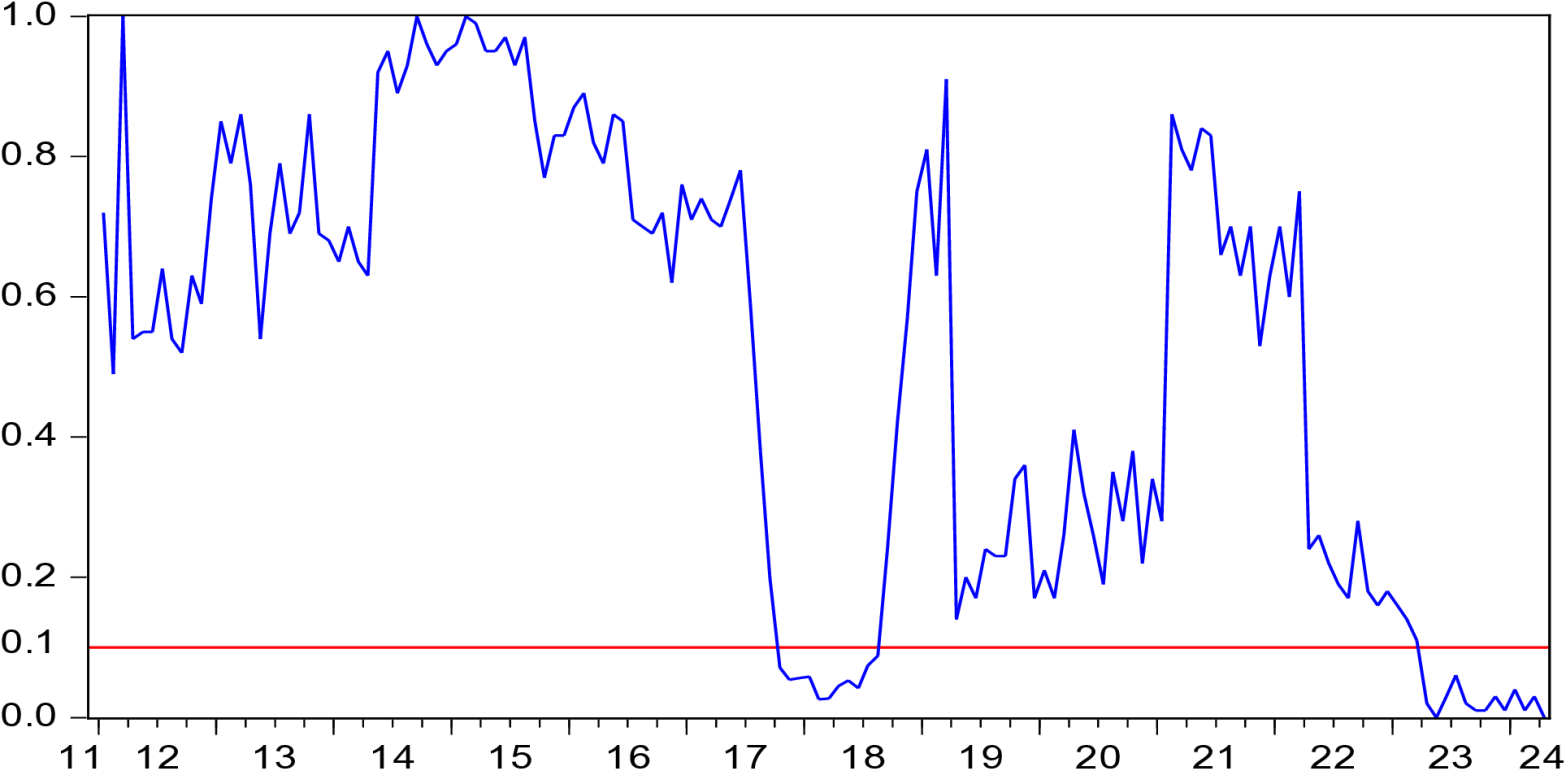
*p-*values of the rolling-window estimation examining the null that EV is not Granger cause of HP.

**Fig 6:**
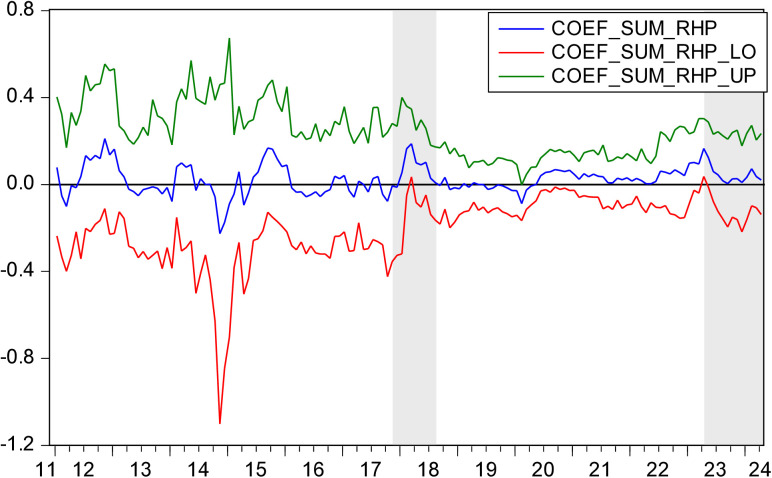
The coefficients for the effect of EV on HP.

Fig 5 displays *p*-values indicating the hypothesis that EV does not Granger cause HP. Fig 6 depicts the direction of influence from EV to HP. Figs 5 and 6 show that in the periods 2017M10-2018M08 and 2023M04-2024M04, EV has a positive influence on HP.

During the periods of 2017M10-2018M08, both EV and HP showed upward trend. In 2018, according to the “2019 Doing Business Report” released by the World Bank, China ranked 46th globally, up 32 places, and was one of the economies with the largest improvement in its business environment. This spurred the emergence of a large number of market entities. As of August 2018, there were 106 million market entities in China, among which newly established ones accounted for 73% of the total. In the first three quarters of 2018, an average of 75,600 new market entities were established every day. Additionally, in 2018, China’s real estate sales area was 148 million square meters, reaching a peak in volume. Sales volume reached 12,639.3 billion yuan, up 14.7%. The positive impact of EV on HP can be explained from three aspects. First, entrepreneurship improves factor productivity through knowledge spillover, technology diffusion, and a driving effect, thus creating more social wealth, enhancing the purchasing power of society as a whole, and positively affecting HP [66]. Second, entrepreneurship not only increases social employment but also improves the income level of practitioners [67]. Higher incomes lead to higher purchasing power, which raises HP [68]. Third, entrepreneurship requires business premises, which drives up demand for real estate, consequently boosting HP.

During the periods of 2023M04–2024M04, both EV and HP showed a downward trend. In March 2023, Silicon Valley Bank (SVB), which primarily served tech start-ups, collapsed due to insolvency. In the post-pandemic era, the global economic recovery was sluggish and slow. The collapse of SVB further severely undermined confidence in market recovery. In 2023, the Chinese government issued a series of laws, regulations, and policies to lower the threshold for entrepreneurship, reduce entrepreneurial risks, and stimulate entrepreneurial vitality. However, the public’s willingness to start a business had correspondingly weakened. In 2023, 32.73 million new business entities were established in China, up 12.6% year-on-year, marking one of the lowest growth rates in the research period. In addition, in 2023, China’s housing sales area was 1,117 million square meters, down 8.5%. The sales volume of housing was 11.66 trillion yuan, down 6.5%. The National Housing Prosperity Index was below 95, in a very depressed range. During 2023M04-2024M04, the positive influence pathway of EV on HP was the same as that during 2017M10-2018M08.

In short, the results obtained from the bootstrap full-sample method indicate that HP Granger-cause EV, but EV does not Granger-cause HP. However, this conclusion does not hold when the coefficients in the VAR(*p*) model are not constant. To demonstrate this variability, this study utilizes four parameter stability techniques, revealing abrupt structural changes in HP, EV, and the VAR(*p*) system. Subsequently, we employ the more sophisticated sub-sample technique to identify the complex relationship between these two variables. The findings indicate that HP can exert both positive and negative impacts on EV. Conversely, there is exclusively a positive influence of EV on HP.

## Conclusions and policy implications

This paper investigates whether HP promote or inhibit EV by examining the time-varying causal links between these two variables. We find that there is significant uncertainty regarding the relationship between HP and EV. In certain periods, HP may positively influence EV through wealth effects. However, in other periods, HP can also negatively suppress EV through substitution effects. Moreover, the positive impact of EV on HP suggests that EV can contribute to the appreciation of HP.

By adopting a multifaceted approach that considers the complex and time-varying interactions between HP and EV, the government can create a more conducive environment for sustainable entrepreneurial growth. First, the government can leverage this positive relationship by implementing policies that support stable housing market growth, ensuring that the wealth effect does not lead to speculative bubbles that could undermine long-term economic stability. To establish a multi-tiered transaction cost regulation mechanism, differentiated value-added tax policies should be implemented for short-term property transactions, with particularly high rates imposed on properties sold within five years of purchase to effectively curb speculation by increasing transaction costs. Concurrently, a progressive property tax system targeting multiple-property owners should be introduced to discourage investment-driven purchases by raising holding costs. Furthermore, stricter oversight should be exercised over the improper flow of credit into the real estate market, with enhanced monitoring of consumer loans and business loans diverted to property purchases. Financial institutions violating regulations should face substantial fines and restrictions on mortgage lending activities to ensure proper allocation of financial resources. Second, to mitigate the negative substitution effect, the government should implement measures to redirect capital flows from real estate speculation to productive entrepreneurial activities. Establish a property-to-venture capital conversion channel, allowing households to reinvest a portion of proceeds from selling excess properties into government-certified venture funds while exempting capital gains taxes. Provide tax rebates or incentives for individuals and enterprises investing in qualified startups, encouraging long-term capital commitment to innovation-driven businesses. Additionally, implement phased subsidy programs for startups, with gradually increasing support tied to business longevity, helping early-stage ventures overcome critical survival periods. Third, the government should actively promote financial marketization and foster financial development to reduce FC associated with entrepreneurship. Specifically, government needs to enhance financial support for small and micro entrepreneurs. Expansion of entrepreneurial guarantee loans could be implemented by broadening coverage, raising loan ceilings for individuals and small and medium-sized enterprises, and adopting the no principal repayment renewal model to alleviate short-term liquidity constraints. Dedicated credit guarantee instruments may also be introduced to facilitate low-cost financing for promising ventures while reducing lenders’ risk exposure. To accelerate technology commercialization, enhancements to intellectual property valuation and pledge financing mechanisms are proposed, complemented by interest subsidies for qualified intellectual property-backed loans. A pre-risk compensation scheme could be piloted, wherein partial default risks are absorbed by public agencies or policy guarantors to incentivize bank participation.

The present study has certain limitations that warrant further exploration. Notably, our dependent variable, EV, is measured by the number of newly established market entities. Therefore, this research primarily focuses on the occurrence of entrepreneurial behavior rather than the success of entrepreneurial activities. After entering the entrepreneurial market, there are two possible outcomes for entrepreneurs: one is successful entrepreneurship, remaining in the market; the other is failed entrepreneurship, exiting the market. However, our study does not elucidate the impact of HP on the outcomes of entrepreneurial activities. If the specific outcomes of entrepreneurial activities could be differentiated, it would more fully demonstrate the influence of HP on EV. We look forward to future research by relevant scholars to address this shortcoming. In addition, the proposed measures face implementation challenges due to China’s significant regional disparities in housing market dynamics. The substantial divergence between tier-1 cities and lower-tier cities creates a policy dilemma. Uniform national regulations may simultaneously over-constrain cooling markets in smaller cities while proving inadequate to curb speculation in overheated metropolitan markets. This spatial variation suggests that our policy recommendations would require careful local adaptation to account for differing market fundamentals, inventory levels, and household wealth structures across regions.

## Supporting information

S1 Dataset Monthly time-series data of HP, EV and FC. (XLSX)
